# Exercise intervention for rumination: from neural mechanisms to clinical applications

**DOI:** 10.3389/fpsyg.2026.1785621

**Published:** 2026-03-06

**Authors:** Junyu Bai, Jinqiao Zhang

**Affiliations:** School of Physical Education, Shaanxi Normal University, Xi’an, China

**Keywords:** cognitive regulation, exercise intervention, mental health, neural mechanisms, rumination

## Abstract

Rumination is a core modifiable cognitive risk factor for the onset and maintenance of mood and anxiety disorders. Exercise emerges as a safe, accessible, and scalable non-pharmacological intervention with potential to mitigate rumination and enhance mental health. This review synthesizes evidence on the neurobiological mechanisms and clinical efficacy of exercise interventions for rumination. Evidence suggests that exercise modulates activity in the default mode network and PFC-limbic circuits, and promotes the release of key neurotransmitters and neurotrophic factors. Therapeutic outcomes are influenced by exercise modality, intensity, frequency, and individual differences. Integrating exercise with psychotherapeutic or digital tools could produce synergistic effects. Future research requires large-scale, longitudinal trials to elucidate the underlying mechanisms and optimize personalized intervention strategies.

## 1 Introduction

Rumination is a maladaptive cognitive pattern characterized by repetitive and passive focus on negative emotions, personal experiences, and the potential causes and consequences of distress (Zhu et al., 2025). This thinking style is closely associated with the onset and maintenance of various psychological disorders, including depression and anxiety (Bean and Ciesla, 2024; Fang et al., 2024). While established interventions like cognitive-behavioral therapy are effective, their implementation is often limited by challenges related to treatment adherence, cost, and accessibility (Peipert et al., 2022; Koffel et al., 2018; Byford and Bower, 2002). Consequently, there is a clear need to identify more accessible, feasible, and sustainable alternative or adjunctive interventions.

In recent years, exercise has gained attention as a practical, sustainable, and multifaceted non-pharmacological intervention for mental health promotion (Koffel et al., 2018; Fibbins et al., 2018; Rimer et al., 2012). Regular exercise improves physical health and may also positively influence rumination through mechanisms involving enhanced neuroplasticity, reduced stress reactivity, and improved affect (Liang et al., 2021; Baskerville et al., 2023; Liu and Tang, 2024; Wang et al., 2025a). Evidence suggests exercise can regulate rumination directly (e.g., by attenuating ruminative thoughts) and indirectly through mediators like increased psychological resilience and improved emotion regulation (Wang et al., 2025b; Guo et al., 2025; Liu et al., 2024). Reductions in rumination are associated with favorable behavioral outcomes; for instance, exercise may improve sleep quality by alleviating both rumination and depressive symptoms (Liu et al., 2025; Abdollahi et al., 2017).

## 2 Neurobiological basis of rumination

**Figure 1:**
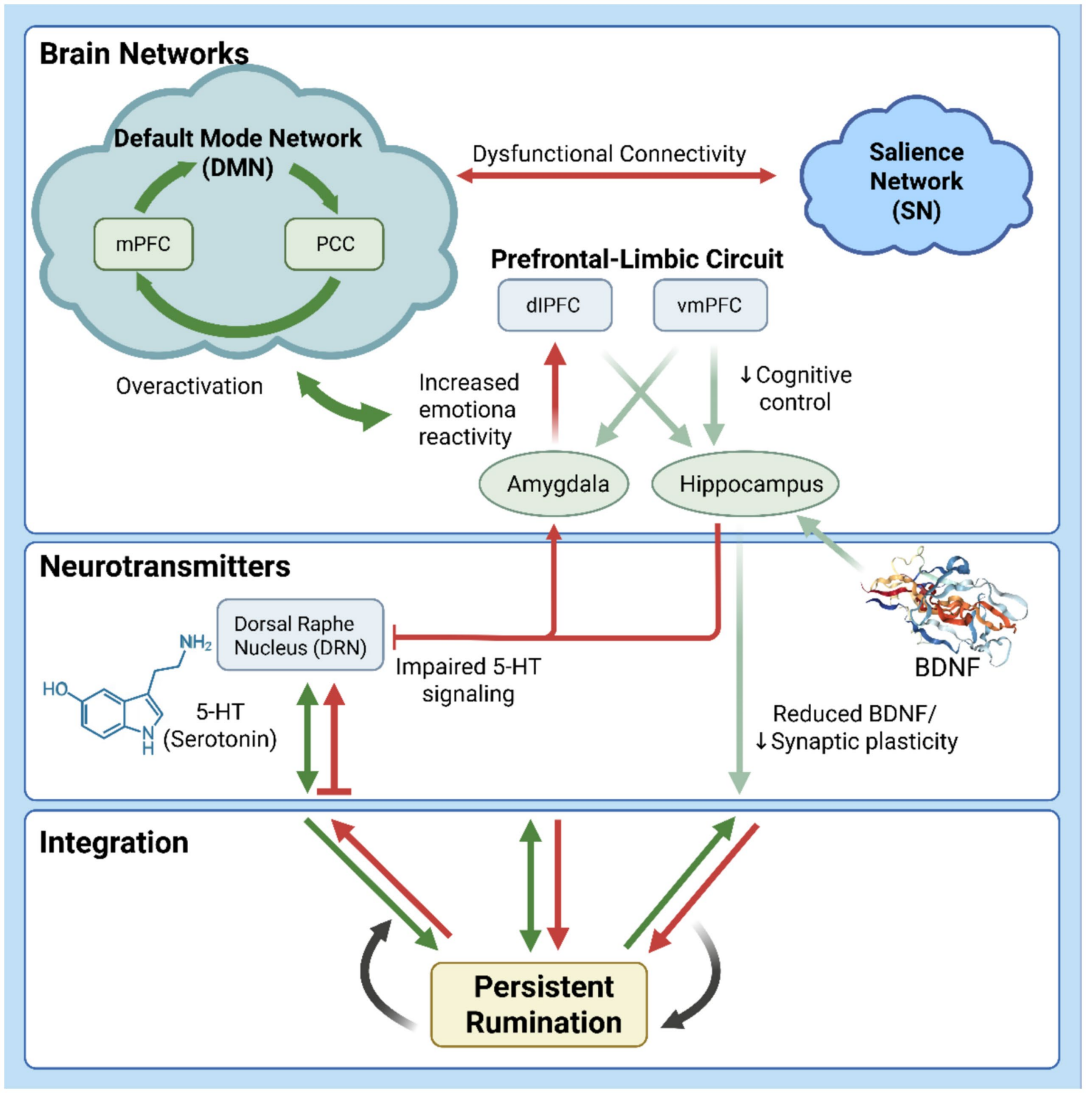
An integrated multi-level framework of the neurobiological mechanisms underlying rumination. DMN, default mode network; SN, salience network; mPFC, medial prefrontal cortex; PCC, posterior cingulate cortex; dIPFC, dorsolateral prefrontal cortex; vmPFC, ventromedial prefrontal cortex; DRN, dorsal raphe nucleus; 5-HT, serotonin; BDNF, brain-derived neurotrophic factor.

The neurobiological mechanisms underlying rumination primarily involve three interrelated domains (Table 1): (1) dysfunction of large-scale brain networks, (2) abnormalities in neural circuit regulation, and (3) impaired molecular signaling. This framework underpins the emergence and persistence of ruminative thinking and offers a basis for understanding how exercise may modulate it (Figure 1).

**Table 1:** Summary of the multi-level neurobiological basis of rumination.

Mechanistic level	Key systems/targets	Major findings and functions	Pathological implications for rumination	References
Brain network level	DMN: core nodes—mPFC, PCC, ACC	Hyperactivation and aberrant connectivity: mPFC/PCC are excessively engaged during negative self-focus. Disrupted intra-DMN and DMN–SN connectivity identified.	Sustains persistent, automatic processing of negative internal information, impeding cognitive disengagement and emotion regulation initiation.	Provenzano et al. (2021), Zhou et al. (2020), Ghaznavi et al. (2023), Yoshimura et al. (2010), Wagner et al. (2015), Qi et al. (2016), Jin et al. (2017), Imperatori et al. (2026), Lydon-Staley et al. (2019), Dégeilh et al. (2018), and Kuehne et al. (2019)
Neural circuit level	PFC-limbic pathways: dlPFC, vmPFC, amygdala, hippocampus	Diminished top-down PFC-limbic control: Reduced dlPFC/vmPFC activity and weakened connectivity with the amygdala. Lower neural signal variability associated with cognitive control.	Underlies cognitive control deficits, resulting in impaired inhibition of negative affect/thoughts and promoting rigid ruminative cycles.	Hiser and Koenigs (2018); Kirkby et al. (2018), Qasim et al. (2023), Hultman et al. (2016), Tully et al. (2014), Vercammen et al. (2012), Andrewes and Jenkins (2019); Myers-Schulz and Koenigs (2012), Waller et al. (2019), Philippi et al. (2022), Zhang et al. (2020), Gao et al. (2023), and Wu et al. (2024)
Molecular/biochemical level	5-HT system (raphe nuclei); BDNF system (Val66Met variant)	Disrupted 5-HT signaling and BDNF abnormalities (e.g., reduced levels or Val66Met polymorphism) impair mood homeostasis and neuroplasticity. Dysregulation of both systems interacts with brain network/circuit alterations.	Establishes biochemical vulnerability, promoting rumination by impairing neurocircuit regulation and plasticity.	Baeken et al. (2021), Soiza-Reilly and Commons (2011), Lv et al. (2022), Kowiański et al. (2018), Erickson et al. (2010), Taliaz et al. (2011), Pattwell et al. (2012), Beevers et al. (2009), Christensen et al. (2008), Zhang et al. (2022), Xu et al. (2016), Franzmeier et al. (2017), and Giles et al. (2017)

### 2.1 Hyperactivation of the default mode network

The default mode network (DMN) shows elevated activity during rest and is integral to self-referential thought and rumination (Whitfield-Gabrieli and Ford, 2012; Provenzano et al., 2021; Zhou et al., 2020). Evidence indicates that rumination is associated with hyperactivation of the DMN, particularly within core regions such as the medial prefrontal cortex (mPFC) and posterior cingulate cortex (PCC) (Ghaznavi et al., 2023). For example, individuals with depression show heightened mPFC and anterior cingulate cortex (ACC) activation when processing negative self-referential information; this activation correlates with symptom severity (Yoshimura et al., 2010). Such abnormal activation is thought to hinder disengagement from negative self-focused stimuli, thereby exacerbating ruminative thought processes (Wagner et al., 2015).

Moreover, atypical PCC activity during affective tasks is frequently linked to deficits in emotion regulation, leading to persistent focus on negative emotions (Qi et al., 2016). Dysfunctional connectivity between the PCC and mPFC may further compromise regulatory capacity (Jin et al., 2017). Beyond impaired intra-DMN connectivity, aberrant connectivity between the DMN and other networks—such as the salience network (SN)—is a central mechanism in rumination (Imperatori et al., 2026). Impaired DMN-SN connectivity may weaken an individual’s capacity for adaptive emotion regulation in response to negative affect, thereby perpetuating ruminative cycles (Lydon-Staley et al., 2019; Dégeilh et al., 2018).

In summary, rumination is characterized by hyperactivation of key DMN nodes and aberrant connectivity both within the DMN and with other networks like the SN. DMN dysfunction thus represents a core neural substrate for rumination in affective disorders.

### 2.2 Dysregulation of the prefrontal–limbic system

Dysfunction in the prefrontal–limbic system is a core neurobiological feature of rumination. This dysfunction centers on a weakened top-down regulatory influence of the prefrontal cortex (PFC) over limbic structures. This impaired circuitry forms the basis for the deficits in cognitive control that characterize rumination.

The PFC—particularly the dorsolateral (dlPFC) and ventromedial (vmPFC) regions—is central to emotion regulation and cognitive control (Kuehne et al., 2019; Hiser and Koenigs, 2018). In contrast, limbic structures like the amygdala and hippocampus are key to emotional processing and memory (Kirkby et al., 2018; Qasim et al., 2023). Rumination involves a failure to inhibit negative affective information, reflecting a deficit in prefrontal control over limbic activity (Hultman et al., 2016).

For example, reduced dlPFC activity is linked to poorer voluntary suppression of negative emotions (Tully et al., 2014; Vercammen et al., 2012). The vmPFC modulates emotional reaction intensity via its regulation of the amygdala (Andrewes and Jenkins, 2019; Myers-Schulz and Koenigs, 2012). Weakened functional connectivity between these regions may exacerbate emotional reactivity (Waller et al., 2019). Additionally, rumination is associated with reduced neural signal variability in the PFC, which may compromise cognitive flexibility and thereby foster repetitive thought (Philippi et al., 2022). Aberrant connectivity between the PFC and limbic structures also impairs emotion regulation capacity and is associated with greater severity of depressive symptoms (Zhang et al., 2020; Gao et al., 2023).

In summary, abnormal activation and functional connectivity within the prefrontal–limbic circuitry undermine cognitive regulation of emotion, predisposing individuals to persistent and inflexible cycles of negative self-focused thought.

### 2.3 Neurotransmitter dysregulation

Dysregulated neurotransmission is a key biological factor in rumination. The serotonin (5-HT) system, central to emotion regulation, is closely linked to affective disturbances and ruminative thought (Wu et al., 2024; Baeken et al., 2021). The dorsal raphe nucleus (DRN), the main source of cerebral 5-HT neurons, modulates emotional responses via its projections to the prefrontal cortex and limbic structures (Soiza-Reilly and Commons, 2011; Lv et al., 2022).

Brain-derived neurotrophic factor (BDNF) is essential for neuronal growth, survival, and synaptic plasticity (Kowiański et al., 2018). Reduced BDNF levels are associated with hippocampal atrophy and functional impairment, which may facilitate rumination (Erickson et al., 2010; Taliaz et al., 2011). Genetic variations, such as the BDNF Val66Met polymorphism, can increase vulnerability to rumination by impairing synaptic plasticity (Pattwell et al., 2012; Beevers et al., 2009).

In summary, abnormalities in the 5-HT and BDNF systems provide a molecular basis for rumination by disrupting emotional homeostasis and neural plasticity, respectively (Wu et al., 2024; Christensen et al., 2008; Zhang et al., 2022). These molecular mechanisms interact with network- and circuit-level dysfunction—for instance, by altering prefrontal–limbic connectivity or DMN activity—thereby promoting rumination (Xu et al., 2016; Franzmeier et al., 2017).

Therefore, the neurobiology of rumination involves a complex, multi-level system characterized by dysregulation across molecular, circuit, and network domains. Understanding their interplay is crucial for elucidating its pathophysiology and for developing targeted interventions.

## 3 Mechanisms underpinning the effects of exercise interventions on rumination

### 3.1 Exercise-induced suppression of default mode network activity

Exercise can reduce rumination, in part, by attenuating hyperactivity in the default mode network (DMN). Recent evidence demonstrates that rumination is associated with increased functional connectivity between the DMN and the central executive network (CEN) (Imperatori et al., 2026). This heightened connectivity correlates positively with general psychopathology. This finding directly implicates the DMN in ruminative processing and suggests that its interaction with the CEN influences broader mental health. Thus, interventions targeting these connections could directly improve psychological well-being.

Exercise enhances cognitive control functions—such as inhibitory control, selective attention, and working memory—that depend on the prefrontal cortex and the CEN. By strengthening these higher-order networks, exercise may improve suppression of DMN activity, thereby reducing rumination (Giles et al., 2017). For instance, exercise improves emotional and cognitive function in individuals with depression and enhances neural efficiency in healthy populations (Gourgouvelis et al., 2017).

In summary, exercise likely modulates rumination through synergistic mechanisms, including enhanced cognitive control and neuroplasticity. These processes may jointly suppress DMN hyperactivity and negative emotion-driven rumination, supporting exercise as a viable emotion regulation strategy.

### 3.2 Exercise-induced enhancement of prefrontal cortex function

In addition to suppressing default mode network activity, exercise can enhance the structural and functional integrity of the prefrontal cortex (PFC). This enhancement improves the capacity for active rumination regulation. The PFC is a key neural substrate for executive function, central to emotion regulation and cognitive control (Friedman and Robbins, 2022; Girotti et al., 2018). At the structural level, exercise increases gray matter volume and attenuates age-related atrophy in the PFC (Soshi et al., 2021; Tamura et al., 2015). These changes provide a stronger neural basis for cognitive regulation.

At the functional level, exercise strengthens connectivity between the PFC and other regions involved in emotion and memory processing, a mechanism linked to reduced rumination (Hanson et al., 2019). For example, enhanced dorsolateral PFC (dlPFC) function is associated with less counterfactual thinking and regret, particularly in individuals prone to self-critical rumination (Allaert et al., 2021). Improved connectivity between the ventromedial PFC (vmPFC) and hippocampus supports the adaptive reorganization of emotional memories, which can reduce negative memory-based rumination (Cowan et al., 2020).

In summary, by promoting both structural plasticity and functional integration of the prefrontal cortex, exercise provides a robust neurophysiological foundation for improved emotion regulation and the inhibition of ruminative thought, highlighting its utility as a targeted intervention for maladaptive cognitive patterns.

### 3.3 Modulation of cognitive and behavioral patterns by exercise

Beyond direct neurobiological effects, exercise reduces rumination by modifying cognitive and behavioral patterns. The underlying mechanisms are multifaceted, including the direct regulation of brain network activity (e.g., suppressing DMN hyperactivity and normalizing DMN–CEN connectivity), the enhancement of prefrontal-mediated cognitive control and neuroplasticity, and endogenous processes such as pain-offset relief that improve affective states (Liang et al., 2021; McFadden et al., 2013; Cao et al., 2016; Suwabe et al., 2021; Vanderhasselt et al., 2017; Vaegter et al., 2020; Harmon-Jones et al., 2019). Together, these mechanisms provide a scientific rationale for exercise as a non-pharmacological emotion regulation strategy.

Integrative interventions combining exercise with other approaches demonstrate these effects. For example, programs like “MAP Train My Brain™” (meditation and aerobic exercise) can modulate functional brain connectivity, improving emotional and cognitive states (Lifshitz et al., 2019) and enhancing psychological well-being (Curlik 2nd and Shors, 2013). Similarly, exercise combined with cognitive training can improve brain network synchrony, reducing rumination in individuals with depression (Shaw et al., 2022).

Even outside structured programs, daily exercise can alter maladaptive cognitive-behavioral patterns. These psychological and behavioral shifts often co-occur with beneficial neurochemical changes. Aerobic exercise promotes hippocampal neurogenesis, while mindfulness and cognitive training support the survival of new neurons (Shors et al., 2014). Such integrated approaches alleviate depressive symptoms and rumination while increasing synchrony in brain networks involved in cognitive control (Alderman et al., 2016). These synergistic effects may be mediated by exercise-induced increases in BDNF, a key factor in depression treatment (Salehi et al., 2014).

Therefore, exercise provides a holistic mind–body experience that reshapes responses to negative internal states. It nurtures mindfulness, enhances behavioral activation, and fosters self-efficacy, forming a complementary intervention pathway to direct neurobiological modulation for reducing rumination.

### 3.4 Effects of exercise on neurotransmitter levels

Exercise modulates rumination by restoring the dynamic balance of neurotransmitter systems and optimizing the neurochemical environment.

Firstly, exercise stimulates endogenous opioid release, which increases pain thresholds and reduces pain perception (Hughes and Patterson, 2020). This effect may involve suppressed inflammatory responses and the release of endogenous analgesics, modulating pain pathways (Hanani, 2025). Notably, reduced negative affect and rumination after acute pain (e.g., cold pressor tasks) are linked to endogenous opioid activation (Harmon-Jones et al., 2019). This system is crucial for emotion regulation: its activation alleviates negative affect and enhances positive emotions via interactions with dopaminergic signaling (Navratilova et al., 2015). Pain relief can also attenuate anger- and sadness-induced rumination, likely due to improved mood after pain offset (Harmon-Jones et al., 2019). Exercise may raise pain thresholds by enhancing central inhibitory function (Belavy et al., 2021).

Secondly, exercise promotes emotional stability and stress resilience by regulating neurotransmitter systems, particularly serotonin (5-HT) (Greenwood, 2019; He et al., 2012). Physical activity counteracts behavioral and neurobiological deficits from sleep deprivation, potentially via 5-HT modulation (de Matos et al., 2025). Conversely, 5-HT deficiency disrupts emotion-related regions like the amygdala, worsening rumination (Godlewska et al., 2012). Moderate aerobic exercise can increase 5-HT levels, mitigating the adverse cognitive effects of sleep loss (Liu and Zhang, 2022).

**Figure 2:**
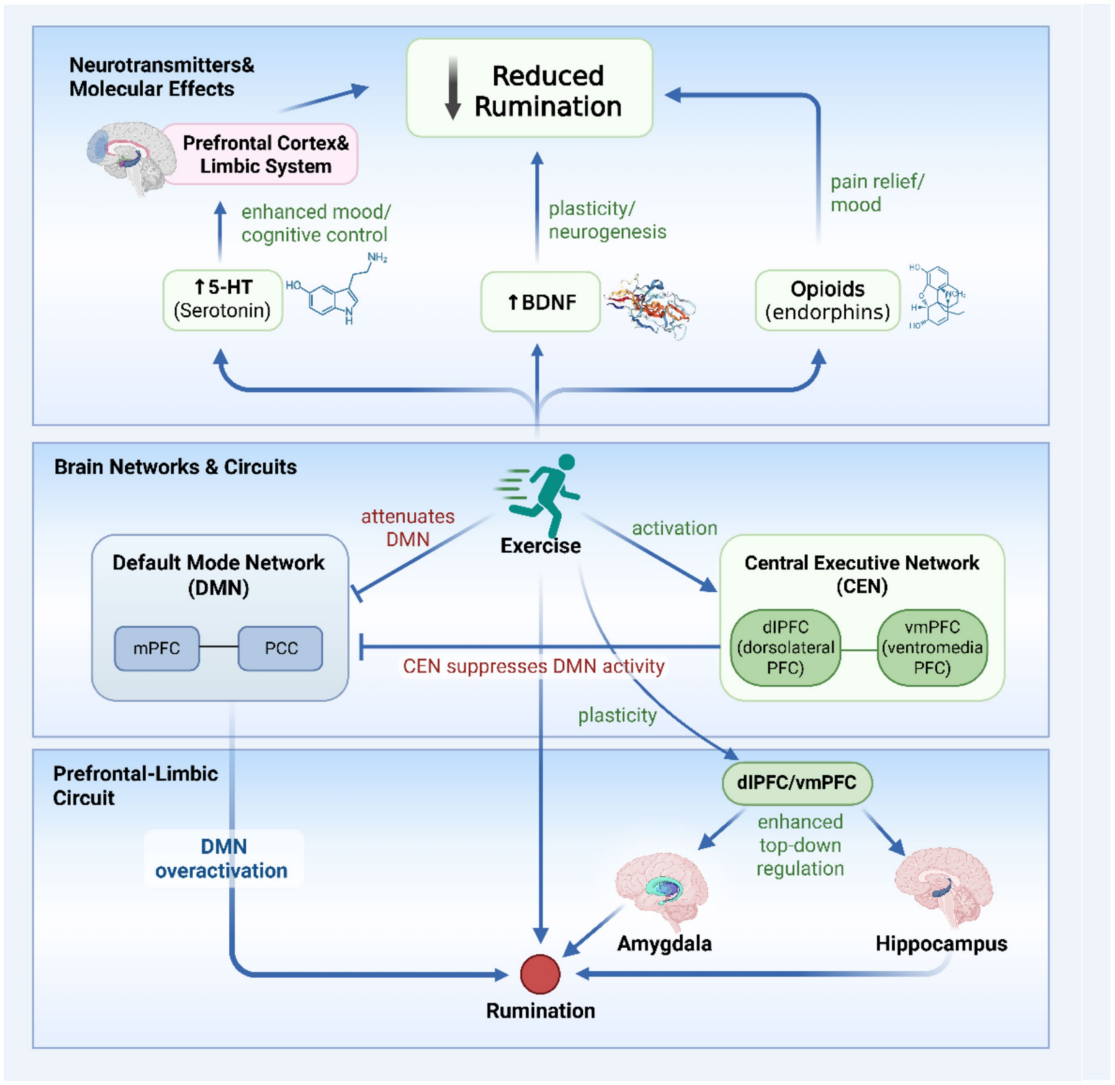
Proposed neurocognitive mechanisms by which exercise modulates rumination.

In summary, exercise reduces rumination not through a single pathway but via the coordinated modulation of multiple neurotransmitter systems. This integrated neurochemical adaptation promotes a more resilient and emotionally stable state, providing a broad biological basis for the efficacy of exercise interventions (Figure 2).

## 4 Comparative efficacy of different types of exercise interventions

**Figure 3:**
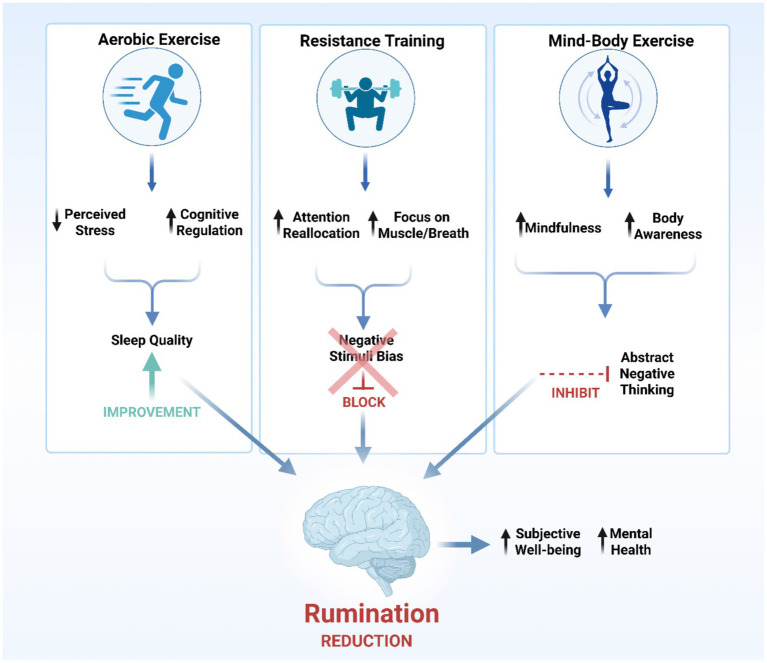
Comparative framework of psychological and cognitive mechanisms by which different exercise modalities reduce rumination. The downward-pointing black arrow denotes “decrease,” and the upward-pointing black arrow denotes “increase” or “enhance.”

The discussed mechanisms outline the general benefits of exercise. However, different exercise modalities, due to their distinct physiological and psychological demands, may influence rumination through different primary pathways. Comparing their efficacy therefore involves examining their dominant regulatory mechanisms (Figure 3).

### 4.1 Association between aerobic exercise and rumination

Aerobic exercise (e.g., running, swimming) regulates rumination with both immediate and sustained effects.

Short-term, single sessions of moderate-intensity aerobic exercise can rapidly reduce state rumination. The “distraction hypothesis” posits that diverting attention from ruminative thoughts and negative emotions to engaging or neutral activities reduces rumination via attentional disengagement from negative cognitions (Nolen-Hoeksema, 1991). Studies show such activity decreases self-reported rumination and facilitates attentional disengagement, supporting the “distraction hypothesis” (Welkerling et al., 2025). These acute benefits may also arise from rapid affective regulation—for instance, speeding recovery from induced negative moods (Bernstein and McNally, 2017a) and improving the use of adaptive strategies like cognitive reappraisal during stress (Bernstein and McNally, 2017b). This can lower the tendency to initiate and maintain rumination.

Long-term, regular aerobic exercise induces persistent cognitive and neuroadaptive changes that reduce vulnerability to rumination. Chronic training improves cognitive function and cerebrovascular regulation (Guadagni et al., 2020) elevates peripheral BDNF levels, and supports neural plasticity (Huang et al., 2021), —all contributing to rumination relief. Furthermore, sustained aerobic activity is linked to a significantly lower incidence of depression (Svensson et al., 2019), a condition closely tied to rumination (McLaughlin and Nolen-Hoeksema, 2011). Thus, the benefits of aerobic exercise are hierarchical: short-term effects focus on affective and attentional regulation, while long-term benefits stem from durable neurocognitive remodeling.

### 4.2 Association between resistance training and rumination

Resistance training provides a distinct intervention pathway for rumination by emphasizing attentional control and behavioral activation, which are processes that require focused attention and goal-directed action.

This exercise modality requires sustained focus on movement execution, breathing, and muscular sensations. This focused attention reduces attentional bias toward negative stimuli, thereby decreasing rumination frequency (Möbius et al., 2018; Lohse and Sherwood, 2012). Integrating breath regulation helps individuals maintain calm under stress, further minimizing ruminative thought (Schwerdtfeger et al., 2019). Such attentional resource redistribution can help break the cycle of persistent focus on negative events (Lask et al., 2021). Moreover, resistance training integrates principles of behavioral activation and self-efficacy enhancement—through goal setting and overcoming physical challenges (Derelioğlu et al., 2025). This structured, goal-oriented activity may buffer the adverse psychological effects linked to rumination (Garner et al., 2025). In summary, the benefits of resistance training for rumination likely stem from the synergistic effects of attentional redistribution and cognitive–behavioral restructuring. However, its independent efficacy, optimal dosage, and long-term effects require further empirical investigation.

### 4.3 Association between mind–body exercise and rumination

Mind–body exercises (e.g., yoga, tai chi) offer potential benefits for psychological well-being through the integration of physical activity and mindfulness. While exercise generally can increase mindfulness (Clark et al., 2015), mind–body practices systematically combine movement with meditation, breath control, and guided intention, thereby cultivating mindfulness more effectively (de Bruin et al., 2020). This enhanced mindfulness improves emotion regulation and subjective well-being (Song et al., 2025).

A core feature of mind–body exercise is its direct focus on the repetitive, negative thinking about the past or future that characterizes rumination (Schmalzl et al., 2014). These practices anchor attention on present-moment bodily sensations. This trains attentional regulation (Wang et al., 2023; Gothe et al., 2016) and facilitate emotion regulation (Zou et al., 2018), enabling a shift from abstract thinking to concrete somatic experience, thereby reducing rumination (Forstmann et al., 2012).

Typically, the benefits of mind–body exercise involve sequential psychological mechanisms. Evidence indicates that physical activity improves well-being by reducing rumination and increasing mindfulness (Wang et al., 2025a). This sequential process clarifies the rationale for mind–body interventions: physical activity reduces negative thinking, while the integrated mindfulness component consolidates cognitive gains (Norouzi et al., 2024; Bing-Canar et al., 2016). Thus, mind–body exercise constitutes an integrated cognitive–behavioral intervention for alleviating rumination in conditions like depression (Alderman et al., 2016), functioning through combined behavioral and cognitive mechanisms.

## 5 Moderating factors of exercise interventions

**Figure 4:**
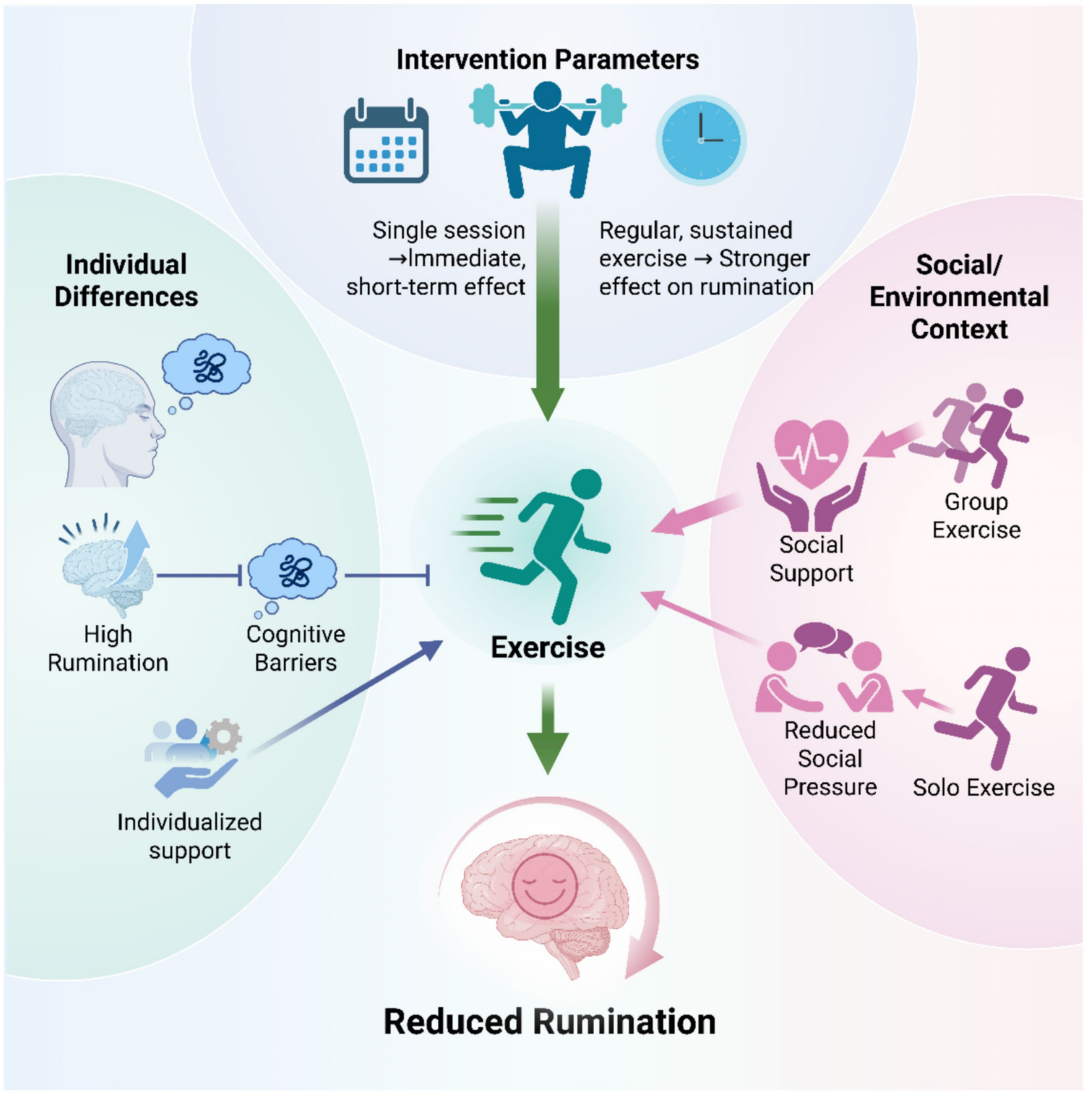
Key moderating factors influencing the efficacy of exercise interventions for rumination.

The efficacy of exercise interventions is moderated by multiple factors, including individual characteristics, intervention parameters, and context. Clarifying these moderators is essential for designing personalized strategies (Figure 4).

### 5.1 Individual factors: interindividual differences

Individual differences significantly moderate the effect of exercise on rumination and mental health, with baseline rumination being a key moderator. Firstly, rumination can itself hinder exercise participation. For instance, negative expectations about physical activity can reduce motivation in children (Ling et al., 2023). This implies that for individuals with high baseline rumination, initial interventions should focus on addressing cognitive barriers, not merely promoting exercise volume.

Furthermore, rumination mediates the relationship between physical activity and mental health: exercise reduces rumination, which is associated with fewer internalizing problems (McLaughlin and Nolen-Hoeksema, 2012; Huang et al., 2025). Therefore, the pathway through which exercise reduces distress by lowering rumination is particularly relevant for individuals with high ruminative tendencies (Cook et al., 2019).

In summary, assessing baseline rumination is crucial for designing exercise interventions. For high-rumination individuals, integrating cognitive strategies to modify maladaptive thinking can improve both adherence and intervention efficacy.

### 5.2 Intervention parameters: optimization of intensity and dosage

The optimal exercise intensity and frequency for reducing rumination are not yet fully defined, but evidence from psychological mechanisms offers a basis for optimizing interventions.

The psychological benefits of exercise are often mediated by sustained changes in cognitive processes, such as reduced rumination and increased mindfulness. These cognitive changes are linked to improved subjective well-being and sleep quality (Song et al., 2025). This connection highlights the need for exercise interventions to be consistent and regular. The daily fluctuation of state mindfulness and its delayed benefits (Mirabito and Verhaeghen, 2023) further indicate that lasting changes in stable cognitive traits require long-term, regular practice. For example, an 8-week program combining aerobic exercise and mindfulness yoga improved emotion regulation and mindfulness (Zhang et al., 2019). This suggests that sustained engagement can effectively cultivate mindfulness and promote mental health.

To meaningfully reduce rumination—a relatively stable cognitive trait—interventions likely require sufficient duration and frequency to allow the repeated activation of positive psychological states. Although a single session of moderate-intensity exercise can acutely lower rumination (Welkerling et al., 2025), regular, long-term exercise is best for maintaining these benefits (Bernstein and McNally, 2018).

Future studies should therefore establish the dose–response relationship between exercise parameters and rumination reduction to guide precise, individualized prescriptions.

### 5.3 Implementation context: social and environmental factors

The efficacy of exercise interventions for reducing rumination is also moderated by their social and environmental context. Group-based and individual exercise operate through distinct psychosocial mechanisms, leading to different outcomes.

Group exercise provides interpersonal support and social embeddedness. Participation enhances social–emotional skills and peer acceptance in adolescents (Zeng et al., 2025; Lee et al., 2019; Steggerda et al., 2024), and alleviates loneliness in older adults (Sebastião and Mirda, 2021). Sustained engagement in team sports significantly reduces perceived loneliness among youth, with the collective nature of such activities conferring additional health benefits (Owen et al., 2024). This enhanced social connectedness can buffer against rumination triggered by social isolation or comparison (Flett et al., 2024; Kokici et al., 2023).

In contrast, individual exercise offers a psychologically safe space for those with high social anxiety, allowing them to avoid social evaluation and regulate emotions autonomously (Hudd and Moscovitch, 2022). Such settings may also reduce stress and rumination related to social comparison (Stark, 2024). Indeed, upward social comparison can trigger rumination in adolescents (Gu et al., 2022; Burnell et al., 2024), potentially affecting their psychological engagement in physical activity (Qi et al., 2024). This highlights the importance of low-pressure environments in mitigating rumination.

Therefore, the exercise setting should be tailored to individual needs. For those sensitive to social comparison, non-competitive individual or supportive small-group formats may be preferable. Aligning the environment with an individual’s psychosocial profile is essential to maximize the mental health benefits of exercise.

## 6 Clinical applications and future directions

### 6.1 Practical application of exercise interventions in patients with depression

Exercise is a key non-pharmacological strategy in the clinical management of depression. Evidence shows that physical activity reduces the frequency and intensity of rumination, thereby alleviating depressive symptoms through multiple mechanisms (Chen et al., 2025).

Firstly, exercise rapidly improves mood and reduces perceived stress, providing an immediate way to interrupt the negative cycles characteristic of depression (Mu et al., 2024; Mu et al., 2025). Second, it enhances cognitive control and mindfulness, helping individuals notice and disengage from repetitive negative thoughts (Jiang et al., 2025; Zhang et al., 2023). These cognitive and affective changes reduce rumination (Ye et al., 2022) and improve sleep quality by lessening rumination-related sleep disturbances (Yang and Lei, 2025)—creating a positive feedback loop that supports recovery.

When prescribing exercise for depression, clinicians should consider not only frequency, intensity, and type (Xie et al., 2021; Heissel et al., 2023), but also strategies to disrupt negative cognitive cycles. For example, integrating exercise with cognitive-behavioral techniques can help patients identify and modify ruminative thoughts during activity (Sun et al., 2018).

Future clinical practice should develop more structured and individualized exercise protocols that maximize antidepressant effects by specifically targeting rumination and related cognitive processes.

### 6.2 Synergistic effects of combined exercise and psychotherapy

Integrating exercise interventions with psychological therapies—particularly cognitive behavioral therapy (CBT)—may yield synergistic benefits for individuals with depression. Exercise offers “bottom-up” physiological improvements, including enhanced neuroplasticity and the modulation of neurotransmitter systems, leading to symptom alleviation (Heinzel et al., 2018; Gujral et al., 2017). Additionally, exercise contributes to increased self-efficacy (Schulz et al., 2012). In contrast, CBT operates through “top-down” strategies such as cognitive restructuring and behavioral activation, which reduce rumination (Stenzel et al., 2025) and increase psychological flexibility and emotion regulation, further decreasing both rumination and avoidance behaviors (Yasinski et al., 2020).

The combined application of these “bottom-up” and “top-down” interventions has the theoretical potential to more comprehensively address the multiple mechanisms underpinning depressive symptoms and other psychological difficulties. Cognitive-behaviorally oriented physical activity (CBPA) has been shown to buffer the adverse effects of rumination on well-being (Garner et al., 2025). Exercise can be incorporated as a form of behavioral activation within the CBT framework (Bourbeau et al., 2020), or integrated into structured exercise–CBT protocols (Clemente-Suárez, 2020), thereby maximizing treatment potency. Such synergistic interventions may be especially beneficial for those who are insufficiently responsive to monotherapy or wish to minimize pharmacological dependence.

Future research should employ randomized controlled trials to directly compare the long-term efficacy and underlying mechanisms of combined interventions versus single-modal treatments.

### 6.3 Potential of technology-assisted interventions

Technology-assisted tools can enhance exercise interventions through three main avenues: precise assessment and monitoring, personalized intervention and feedback, and multimodal data integration within a closed-loop system. Wearable devices and mobile health applications have improved the feasibility, adherence, individualization, and outcome evaluation of exercise-based interventions (Stenzel et al., 2025; Yasinski et al., 2020; Bourbeau et al., 2020; Clemente-Suárez, 2020; Rehman et al., 2017b; Rehman et al., 2017a; Sun et al., 2021; Deka et al., 2025; Paolucci et al., 2024; Balbim et al., 2021; Agley et al., 2024).

For precise assessment, instruments like the Physical Activity-Related Rumination Scale for Children (PARSC) can identify cognitive barriers to exercise participation (Ling et al., 2023). Emerging tools, such as wearable sensors and the Gait-Specific Attentional Profile (G-SAP), enable continuous monitoring of behavioral and physiological markers of rumination (Young et al., 2020; Hardeman et al., 2020; Roxburgh et al., 2019), facilitating individualized evaluation.

Mobile health platforms support tailored interventions by delivering adaptive exercise, mindfulness, or cognitive tasks based on ongoing assessments (Sun et al., 2021; Vandenbogaart et al., 2023). These systems can integrate ecological momentary assessment to synchronously monitor dynamic changes in physical activity, mood, and rumination (Rosenblum et al., 2025; Daniels et al., 2025). This provides real-time insight into the link between exercise and psychological states. Accordingly, app-based systems can deliver personalized prescriptions, mindfulness reminders, and cognitive training modules (Sandkühler et al., 2025). This enables a “monitoring–feedback–adaptation” closed-loop process, which is particularly valuable for specific populations like retired athletes (Iverson et al., 2025; Albishi et al., 2025).

**Figure 5:**
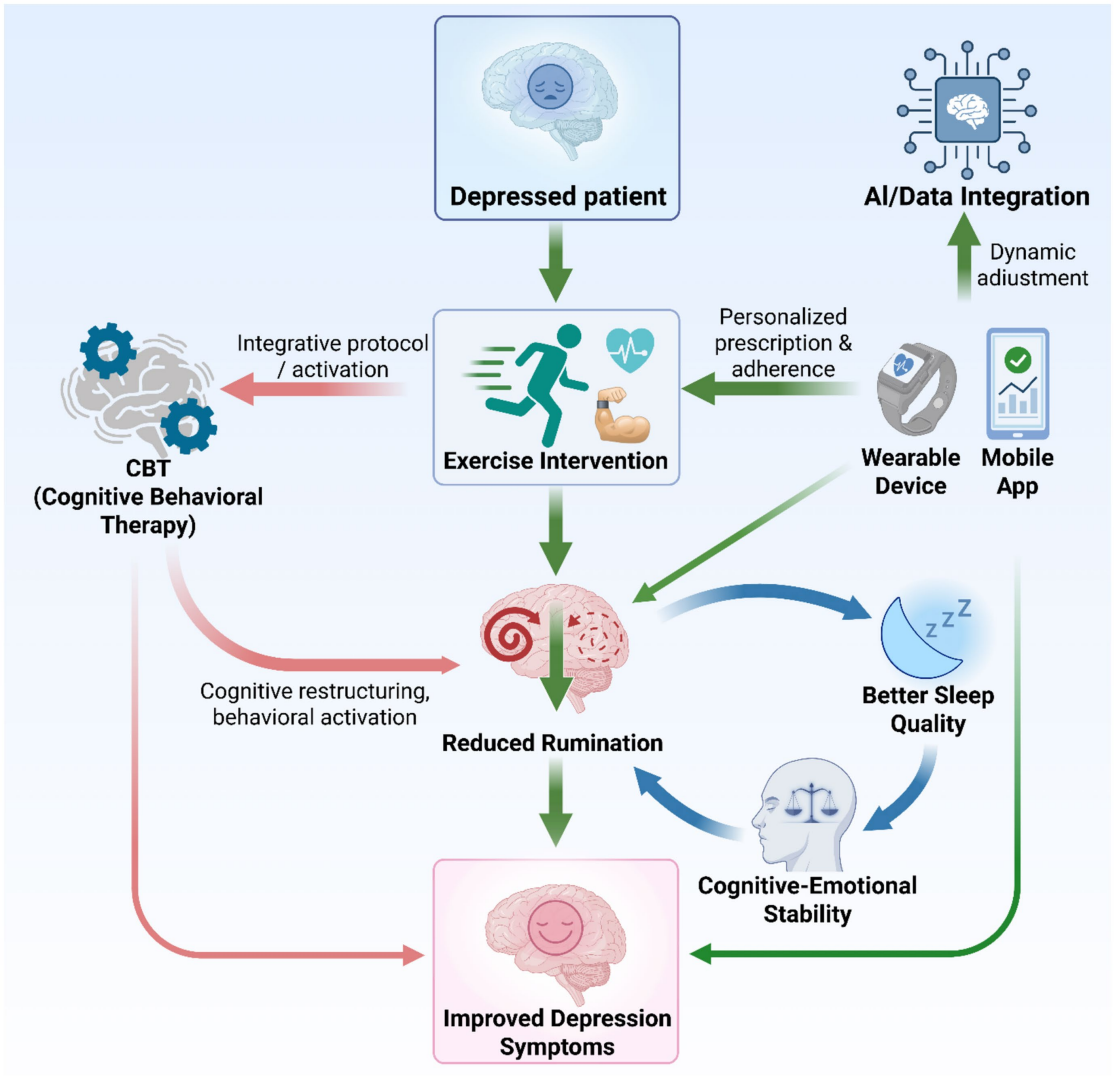
An integrated intervention model for depression combining exercise, psychotherapy, and digital tools.

Future research should validate these technologies across diverse populations, optimize their usability and long-term adherence, and explore integrating artificial intelligence for dynamic, automated intervention adjustments through multimodal data fusion (Figure 5).

## 7 Conclusion

Exercise shows promise as a non-pharmacological intervention for rumination. Evidence indicates its benefits are mediated through complementary mechanisms: neurobiologically, by promoting neurotrophic factor release and modulating prefrontal–limbic circuits; psychologically, by enhancing cognitive flexibility and emotion regulation. However, limitations remain, including sample heterogeneity, poorly standardized protocols, and insufficient evidence on long-term effects.

Intervention efficacy is moderated by exercise type (aerobic, resistance, mind–body), intensity, frequency, and individual differences. For example, high-intensity interval training (HIIT) may improve executive function, while mind–body practices like yoga show specificity in emotion regulation. Future work should develop personalized prescriptions by integrating biomarkers and digital tools.

Practically, exercise interventions are accessible and low-risk, but long-term adherence is challenging. To improve sustainability, combined models (e.g., exercise with cognitive or mindfulness training) and community-based delivery warrant further exploration.

In summary, exercise is a viable adjunctive strategy for mental health. Future research requires large-scale, rigorous trials to clarify mechanisms, establish guidelines, and inform clinical implementation.
